# The Effects of Craniofacial Muscle Contractions on the Formerly Vagus Nerve Somatosensory Evoked Potentials

**DOI:** 10.7759/cureus.83802

**Published:** 2025-05-09

**Authors:** Mario A Mosquera, Juan S Leon-Ariza, Angelo Fonseca, Daniel S Leon-Ariza, Samuel Iglesias, Jorge C Mora, Fidias E Leon-Sarmiento

**Affiliations:** 1 Surgical Neurophysiology, Baptist Health, Miami, USA; 2 Family Medicine, Saint Francis Hospital, Wilmington, USA; 3 Neurosciences, Mediciencias Research Group, Bogota, COL; 4 Neurology, Ochsner Medical Center, New Orleans, USA; 5 Translational Medicine, Florida International University, Herbert Wertheim College of Medicine, Miami, USA; 6 Neurology, Surgical Neurophysiology Unit, Medical University of South Carolina, Charleston, USA

**Keywords:** afferents, brainstem, eeg, evoked potential, vagus nerve

## Abstract

Objective: Historical studies reported that electrical stimulation applied over vagus nerve (VN) afferents from the tragus of the human ear-induced skull responses labeled as the vagus nerve somatosensory evoked potential (VSEP). Miscellaneous results acquired from healthy and diseased populations suggested that the origin of the VSEP might not correspond to brain neural activity, but rather to unwanted electromyographic oscillations. Our objective is to definitively demonstrate that scalp recordings labeled as the formerly VSEP (fVSEP) are the expression of muscle activity surrounding recording electrodes.

Methods: Using surface electrodes, we electrically stimulated the right ear tragus of five healthy male individuals (mean age: 44 ± 12 years) at 2, 4, 6, 8, and 10 mA, respectively. We recorded the VSEP from the skull of participants while they were relaxed and during controlled voluntary craniofacial muscle movements.

Results: Increasing the stimulus intensity significantly paralleled the increase of the motoneuronal recruitment of the fVSEP during relaxed conditions (eyes open: p = 0.04; eyes closed: p = 0.03). Likewise, voluntary craniofacial muscle contractions significantly modified the duration (p < 0.01) and amplitude (p = 0.013) of the fVSEP.

Conclusions: Our results indicate that the fVSEP is the graphical expression of depolarization and repolarization overflow of signals happening at a distance from the source, as well as the electromyographic expression of unwanted muscle oscillations exerted by craniofacial muscle events registered beyond the point of recording. Therefore, the fVSEP should no longer be considered a brain somatosensory evoked potential nor a measure of autonomic function.

## Introduction

Sensory nerve afferents from the vagus nerve (VN), one of the 13 cranial nerves, innervate the tragus of the human ear [1,2]. These afferents include A-alpha, A-beta, A-delta, and C fibers [3,4]. Electrical stimulation of these sensory afferent fibers in the ear has been employed to induce brain-evoked potentials in humans [5,6]. The skull responses elicited by stimulating the nerve afferents of the human tragus have been termed the vagus nerve somatosensory evoked potential (VSEP) [5,6]. However, nearly two decades of research investigating the origin of VSEP have yielded mixed and inconsistent results in both healthy individuals and participants with neurological disorders. These findings suggest that cranial recordings previously attributed to VSEP may not reflect brain neuronal activity but rather electrophysiological artifacts [3,4,7,8].

To address this controversy, we recently reviewed the existing VSEP literature and presented evidence from human subjects supporting a nonneuronal origin of the VSEP. Additionally, we proposed an alternative interpretation of the VSEP based on volume conduction neurophysics principles [3,4]. Our previous findings also revealed a hyperdirect brain somatosensory pathway following tragus stimulation, which we termed the earliest vagus-evoked responses by stimulating the tragus (EVEREST) [3,4,9].

In this study, we aim to definitively demonstrate that scalp recordings previously identified as VSEP, now referred to as the formerly VSEP (fVSEP), result from muscle activity surrounding the recording electrodes rather than true brain-evoked potentials. This overlooked factor has contributed to significant variability in recorded responses, misleading clinical and pathophysiological interpretations in prior research.

To bridge this gap in knowledge, we hypothesize that fVSEP is not a true brain somatosensory-evoked potential but rather the electrophysiological signature of craniofacial muscle activity induced by electrical stimulation of the tragus. We further predict that increasing the intensity of the electrical stimulation applied over the tragus will proportionally increase craniofacial muscle activity surrounding the electrodes used to record the fVSEP.

To test our hypothesis, we will apply electrical stimulation to the tragus of healthy participants, both at rest and during controlled craniofacial muscle contractions. This study aims to resolve longstanding debates regarding the origin of the fVSEP and to redefine our understanding of somatosensory evoked potentials elicited by tragus stimulation.

## Materials and methods

Five healthy male individuals (mean age: 44 ± 12 years) were studied using the protocol previously described in detail by our group [3,4]. In brief, we recorded skull responses using the International 10-20 system. Standard head bipolar montages (Cz-C4 and C6-T4) were prepared with surface electrodes placed over the scalp ipsilateral to the stimulation site. These electroencephalogram (EEG) montages were selected based on published findings indicating that T4 is the most sensitive electrode for detecting the fVSEP [3,4].

Electrical stimulation was applied over the right tragus (cathode inside, anode outside) using surface electrodes. The stimulus duration was 0.1 ms, with a stimulation frequency of 2.79 Hz. Impedance was maintained below 2 kΩ. The ground electrode was placed over the ipsilateral shoulder.

Each participant underwent the following six conditions: 1) relaxed with eyes open, 2) relaxed with closed eyes, 3) contracting the orbicularis oculi (OOc) muscles at approximately 50% of maximal muscle effort, 4) contracting the OOc muscles at 90% of maximal effort, 5) contracting the masseter muscles at approximately 50% of maximal effort while keeping the eyes open, and 6) contracting the masseter muscles at 90% of maximal effort while keeping the eyes open.

The right abductor digiti minimi muscle served as a control for unwanted voluntary muscle activity. Participants were trained on the same day of the study using visual and auditory feedback to learn how to achieve 50% and 90% muscle contractions.

For conditions 1 and 2, five blocks of 10 simulations each were applied at 2, 4, 6, 8, and 10 mA [3,4]. To prevent potential habituation, each block was performed every 30-60 seconds [10]. For conditions 3-6, which involved muscle contractions, 10 stimulations at 10 mA were applied. The effects of muscle contractions on the fVSEP were studied only at 10 mA to avoid Type II statistical errors due to potential fatigue from prolonged muscle contractions. Additionally, this stimulus intensity is considered safe for humans [11].

Since right tragus stimulation may modulate heart rate [12], surface electrodes were also placed over the precordial region to record the heart R-R interval of the electrocardiogram before and during tragus electrical stimulation.

After receiving a thorough explanation of the study, all participants provided written informed consent. The study protocol was reviewed and approved by the ethics committee of the Mediciencias Research Group and was conducted in accordance with the principles of the Declaration of Helsinki. No participants were compensated for their participation.

Statistical analysis

Responses were stored on a hard drive for offline signal processing and analysis. Data were log-transformed before analysis to mitigate bias from skewed values. The amplitude and duration of the fVSEP were assessed, while latency was not considered due to known technical reasons [13]. EVEREST latency, amplitude, and duration were calculated as described elsewhere [3,4].

A two-sample comparison of the R-R interval was performed using paired t-tests. The effects of stimulus intensity on fVSEP input-output motoneuron recruitment during the eyes-open and eyes-closed conditions were examined using analysis of variance (ANOVA) for correlated observations [14]. Additionally, a one-way repeated-measures ANOVA was conducted to analyze the effects of craniofacial muscle activation on the amplitude and duration of the fVSEP. Further pairwise comparisons using Bonferroni correction were applied to the ANOVA data to identify specific differences in amplitude and duration across craniofacial muscle state conditions.

Because craniofacial muscle status could have influenced the measurements, we collapsed the data from resting and active muscle conditions, computed the mean values of amplitude and duration, and examined correlation coefficients (r) between these two conditions. The effect size was calculated by subtracting the mean amplitude obtained at rest from that during voluntary muscle contraction and dividing the result by the pooled standard deviation. An effect size between 0 and 0.3 was considered small, between 0.3 and 0.7 moderate, and greater than 0.7 large. Statistical significance was set at p < 0.05.

## Results

None of the participants experienced any visible side effects during or up to 30 minutes after electrical stimulation of the right ear tragus. Compared to baseline, the R-R interval did not change during electrical stimulation while participants were at rest with their eyes open (t = 0.22, p = 0.41), eyes closed (t = 0.39, p = 0.35), or when performing voluntary craniofacial muscle contractions (t = -1.5, p = 0.44).

**Figure 1:**
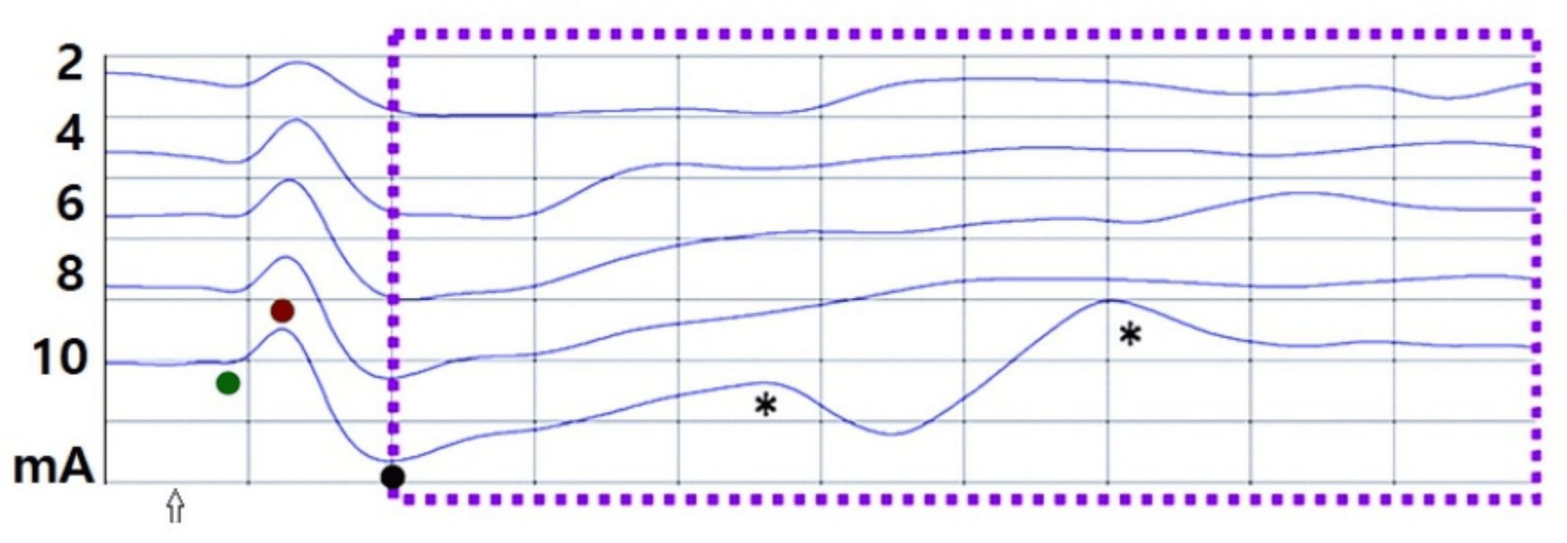
EVEREST and VSEP at rest Recordings were obtained at Cz-C4 in a 51-year-old man with eyes open. The fVSEP (purple dashed lines) was induced by stimulating the ipsilateral tragus at 2, 4, 6, 8, and 10 mA. Note that the shape of the fVSEP changed unevenly with increasing stimulus intensity Asterisks indicate waveforms compatible with A-beta and C-fiber activity. EVEREST was observed at all stimulus intensities and appeared earlier than the fVSEP The arrow indicates stimulus delay. The Y-axis represents amplitude, and the X-axis denotes latency Horizontal: 2 ms/div, vertical: 10 mV V1: green, V2: red, V3: black, fVSEP: formerly vagus nerve somatosensory evoked potential, EVEREST: Earliest Vagus-Evoked Responses by Stimulating the Tragus

Electrical stimulation of the right tragus induced responses with varying waveforms, collectively labeled as the fVSEP. Occasionally, more than one fVSEP waveform was recorded while participants were in a relaxed state during stimulation (Figure 1).

**Figure 2:**
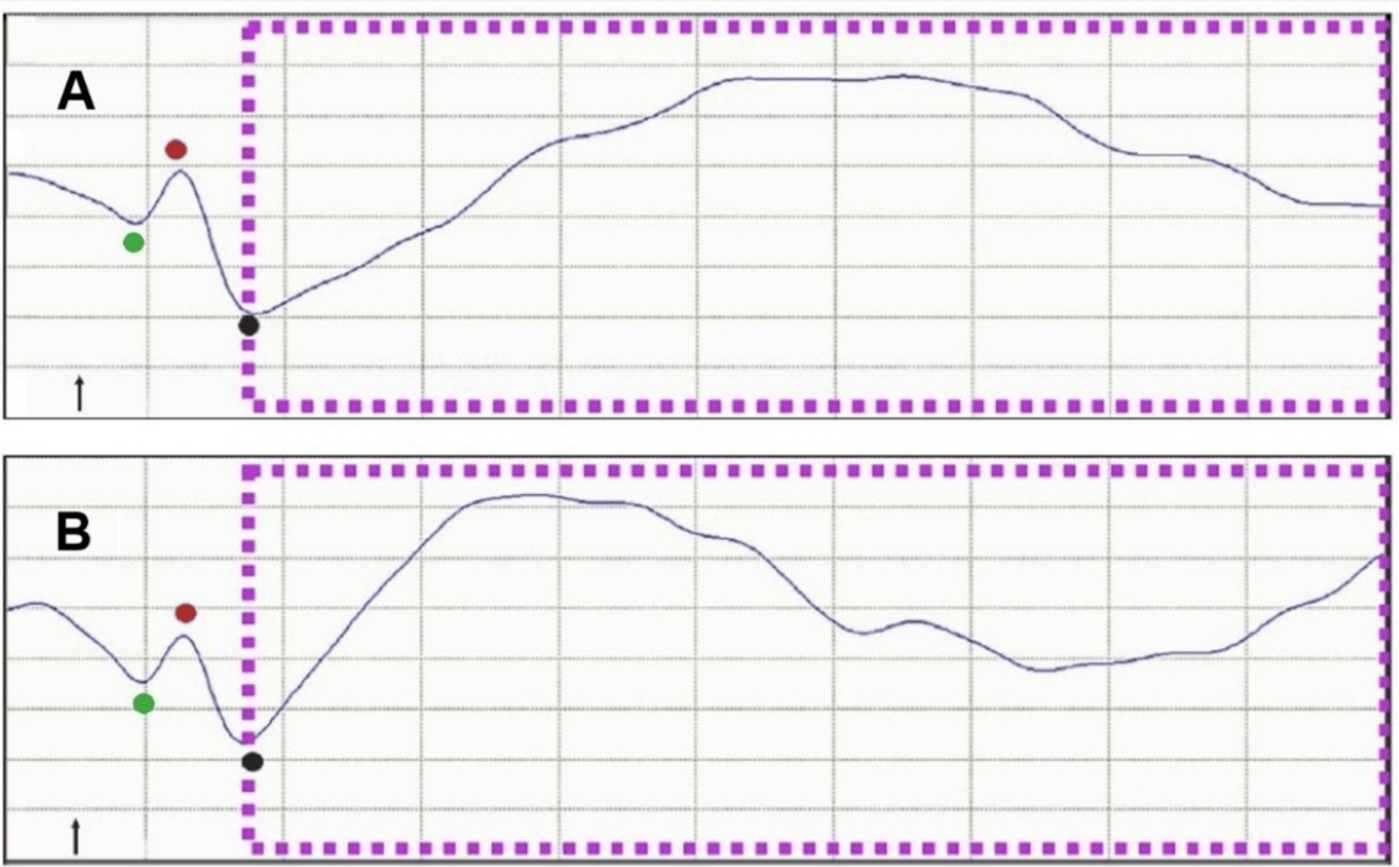
EVEREST, VSEP, and orbicularis oculi muscle contractions Recordings were obtained at C6-T4 in a 47-year-old man performing 50% (A) and 90% (B) of maximal voluntary orbicularis oculi contraction. The fVSEP (purple dashed lines) was induced by applying a 10-mA stimulus to the ipsilateral tragus. Note that the amplitude and duration of the fVSEP waveform varied with muscle effort. EVEREST appeared earlier than the fVSEP and showed a mild decrease in amplitude during stronger muscle contractions The arrows indicate stimulus artifact Horizontal: 2 ms/div, vertical: 15 mV The Y-axis represents amplitude, and the X-axis indicates latency V1: green, V2: red, V3: black, EVEREST: earliest vagus-evoked responses by stimulating the tragus, VSEP: vagus nerve somatosensory evoked potential, fVSEP: formerly vagus nerve somatosensory evoked potential

Distortions in the shape and waveform of the fVSEP were observed in both territorial and extraterritorial craniofacial muscles located far from the stimulation site (e.g., Cz-C4 recordings), and these distortions were related to the magnitude of muscle contraction (Figure 2).

**Figure 3:**
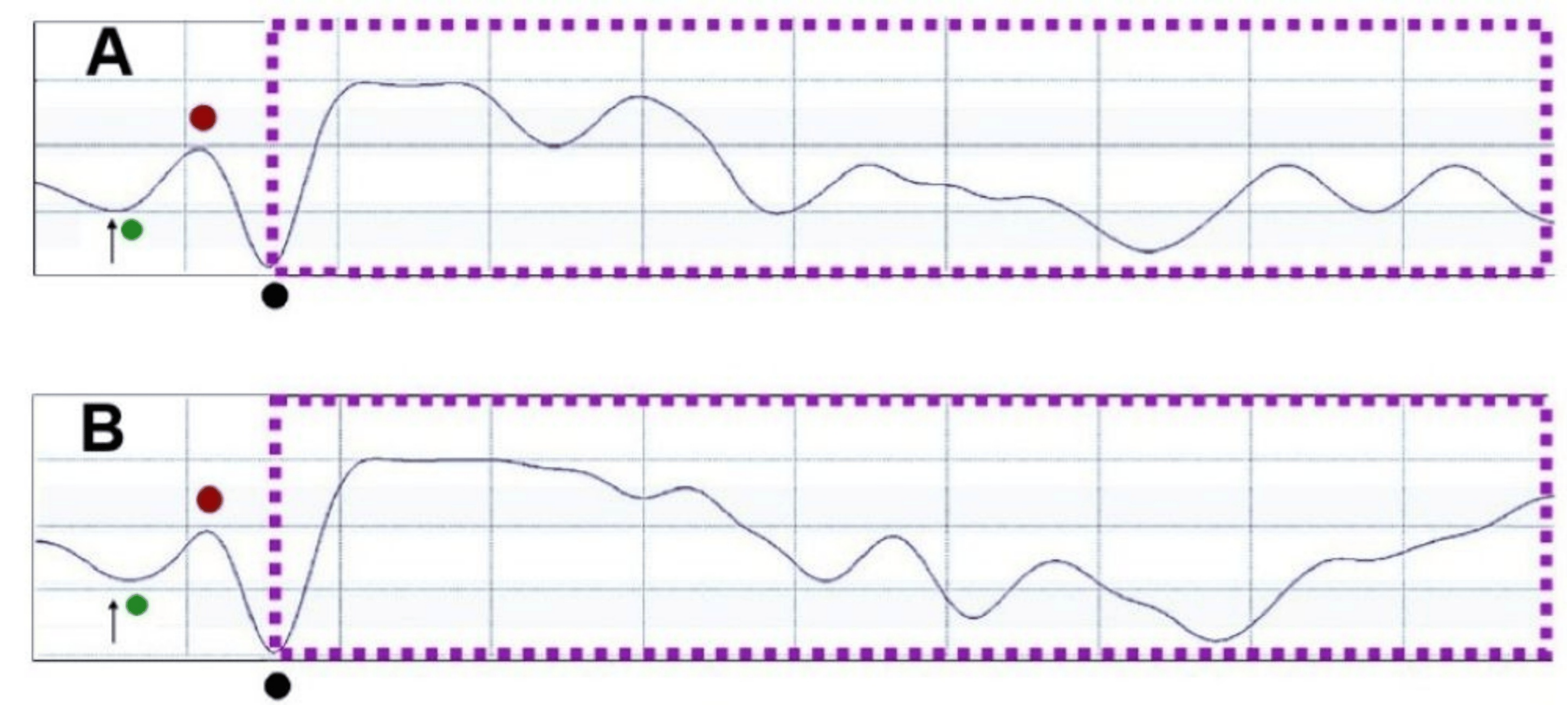
EVEREST, VSEP, and masseter muscle contractions Recordings were obtained at C6-T4 in a 31-year-old man performing 50% (A) and 90% (B) of maximal voluntary masseter muscle contraction. The fVSEP (purple dashed lines) was induced by applying a 10-mA stimulus to the ipsilateral tragus. Note that the amplitude and duration of the fVSEP waveform varied with muscle effort. EVEREST appeared earlier than the fVSEP and showed a mild decrease in amplitude during stronger muscle contractions The arrows indicate stimulus artifact Horizontal: 2 ms/div, vertical: 20 mV The Y-axis indicates amplitude, and the X-axis represents latency V1: green, V2: red, V3: black, EVEREST: earliest vagus-evoked responses by stimulating the tragus, VSEP: vagus nerve somatosensory evoked potential, fVSEP: formerly vagus nerve somatosensory evoked potential

**Figure 4:**
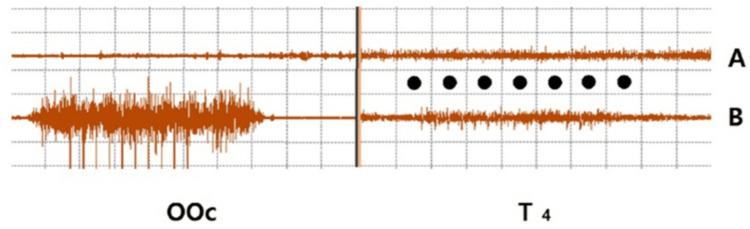
Representative OOc and temporalis (T4) surface EMG (dots) Ten electrical stimulations at 10 mA each were applied to the right tragus of a 47-year-old man while he gently closed his eyes (A) and while performing a 50% maximal OOc muscle contraction (B). EMG activity was minimal in the OOc but noticeable over T4 when the participant received electrical stimulation with eyes closed (A). EMG activity became more pronounced in T4 during voluntary muscle contraction (B) Horizontal: 10 ms/div, vertical: 100 mV The Y-axis represents amplitude, and the X-axis represents the duration of electromyographic activity OOc: orbicularis oculi, EMG: electromyogram

**Figure 5:**
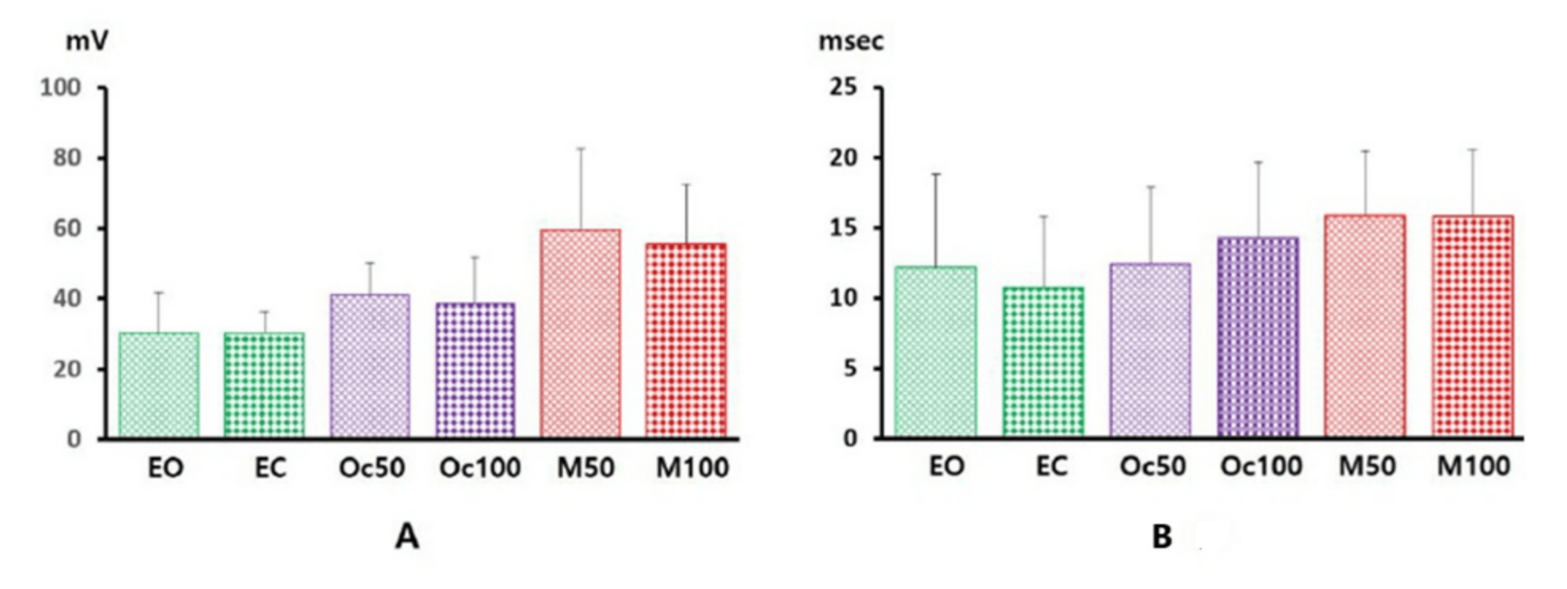
Amplitude (A) and duration (B) values of the fVSEP Amplitude (Y-axis, in mV) and duration (Y-axis, in ms) are represented as thick vertical bars, with standard deviation indicated by thin vertical lines. Data were obtained following electrical stimulation (10 mA) applied to the right tragus of the human ear and recorded under different muscle state conditions (X-axis) Data are expressed as mean ± SD EO: eyes open, EC: eyes closed, Oc50: orbicularis oculi muscle contracted at 50% of maximal effort, Oc100: orbicularis oculi muscle contracted at 100% of maximal effort, M50: masseter muscle contracted at 50% of maximal effort, M100: masseter muscle contracted at 100% of maximal effort, fVSEP: formerly vagus nerve somatosensory evoked potential, SD: standard deviation

ANOVA revealed that increasing the electrical stimulation during relaxed conditions significantly enhanced the amplitude recruitment of the fVSEP when participants had their eyes open (degrees of freedom, DF = 4, F = 2.94, p = 0.04) and closed (DF = 4, F = 3.309, p = 0.03). ANOVA also demonstrated a significant effect of stimulus intensity on the duration of the fVSEP recorded with participants' eyes open (DF = 4, F = 3.8, p = 0.01) and closed (DF = 4, F = 4.8, p = 0.01). Notably, fVSEP activity increased when participants performed craniofacial muscle contractions. ANOVA confirmed that muscle state conditions significantly influenced both the duration (F = 41.2, DF = 5, p < 0.001) and amplitude (F = 3.74, DF = 5, p = 0.012) of the fVSEP (Figures 3-5).

Craniofacial muscle status (relaxed versus active) showed strong correlations with fVSEP amplitude (r = 0.75) and duration (r = 0.99). Bonferroni-corrected pairwise comparisons revealed that fVSEP amplitude was significantly modulated by masseter muscle contractions at 50% of maximal muscle effort (p = 0.05, 95% confidence interval, CI: -0.27 to 59.07). Similarly, fVSEP duration was significantly affected at 50% (p = 0.02, 95% CI: 0.28-7.55) and 90% of maximal muscle effort (p = 0.002, 95% CI: 1.34-8.61). The effect size was 0.77.

Additionally, in all participants, we elicited a novel brain-evoked potential, which our group has termed EVEREST [3,4]. Its morphology, shape, and phases fulfilled the criteria for an evoked potential [15]. We identified three distinct EVEREST components, named V1, V2, and V3 (where "V" denotes vagal or vagus). V1 consistently appeared at the nanosecond scale [3,4]. Although this study was not designed to investigate the effects of electrical stimulation or muscle contractions on EVEREST, we observed that at rest, its latency decreased, and its amplitude steadily increased with greater stimulus intensity (Figure 1). However, in some cases, its amplitude decreased with muscle contractions (Figures 1-3). Detailed morphophysiological aspects of EVEREST, including its neural recruitment, brain topography, neural generators, the effects of female hormones, and the nerve fibers involved in eliciting this novel neural response, will be presented elsewhere.

## Discussion

The fVSEP had been induced only under relaxed conditions in awake participants or in surgical patients under the effects of intravenous neuromuscular blockers [3,4]. In this study, we examined the behavior of the fVSEP in a selected sample of awake individuals under both relaxed conditions and active craniofacial muscle modulation [16]. We demonstrated that the fVSEP is the electromyographic expression of craniofacial muscle activity, most likely induced by A-beta and C-fiber stimulation. It becomes evident with increasing stimulus intensity during relaxed conditions and is further augmented by voluntary craniofacial muscle contractions [17]. These findings are characteristic of volume conduction phenomena, which can occur passively in a three-dimensional structure [18,19] or when motoneurons are actively excited, contaminating EEG signals and mimicking brain neuronal activity [20].

According to Euclidean geometry principles, the amplitude of any waveform is directly proportional to a specific portion of the surface area of the stimulated cell membrane and inversely proportional to the square of the distance between the membrane and the recording electrode [21]. Uncontrolled passive and active craniofacial muscle oscillations violate these Euclidean constraints [19], complicating the physiological interpretation of volume conduction phenomena and leading to the misattribution of muscle contraction effects as true neural activity [21,22].

The volume conduction phenomenon observed in our previous studies under nonneuromuscular blocker conditions [3,4] and replicated here in awake participants is fundamentally the expression of cell membrane depolarization and repolarization induced by tragus electrical stimulation, with these processes extending beyond the recording site. This occurs because recording electrodes capture both intended and unintended signals within a three-dimensional structure (e.g., the human body), as explained by the three-dimensional neurophysics-based interpretation of Ohm’s law [20,21]. Indeed, muscle action potentials increase with stimulus intensity and decrease with both radial distance and conductivity [20,23]. Consequently, when transmembrane currents generating action potentials extend beyond the intended recording site, the signals spread volumetrically (e.g., into surrounding muscles) [24], leading to signal overflow at locations distant from the source [3,4]. Due to these unintended body-machine interactions, muscle oscillations induced by tragus stimulation propagated beyond electrode recordings, producing volume conduction effects historically mislabeled as the fVSEP. These findings fully explain the high variability, poor specificity, lack of reproducibility, and heterogeneous characteristics of the fVSEP, as well as its elimination by neuromuscular blockers [16].

The effects of craniofacial muscle contractions, investigated here for the first time in the context of VN afferent somatosensation, provide definitive evidence that the fVSEP is the macroelectromyographic expression of muscle oscillations induced by electrical stimulation of the human tragus. In fact, the effect size obtained confirmed that our results aligned with predicted outcomes. This motoneuronal activity, previously labeled as the fVSEP, significantly increased beyond 2 ms following tragus stimulation when participants contracted their craniofacial muscles at different intensities. Prior human studies have reported that neuromuscular blockers abolished responses recorded beyond two milliseconds after tragus afferent stimulation [16]. Some groups have interpreted these responses as the VSEP, while others have argued that they represented muscle contamination. Our current findings fully resolve these longstanding disagreements. Notably, similar results have been observed in animal models.

In brief, studies in rats have shown that afferent sensory stimulation of the cervical branches of the VN induces muscle activity beginning around 2 ms and lasting up to 20 ms [25,26]. This activity parallels stimulus intensity and correlates with A-fiber stimulation [25,26]. From previously published recordings [17], it can also be inferred that electrical stimulation excites both A-beta and C-bundle fibers [25,26]. Moreover, consistent with our findings, vecuronium infusion suppressed or eliminated cranial electromyographic activity occurring beyond 2 ms [9], while a hyperdirect component recorded within nanoseconds following A-alpha fiber stimulation remained unaffected [25,26]. These translational studies align well with our ability to consistently record the EVEREST response.

The EVEREST signals fulfilled the evoked potential criteria defined by the International Federation of Clinical Neurophysiology [15]. Notably, the latency of EVEREST consistently falls within the nanosecond range following tragus electrical stimulation [3,4], indicating that it represents true hyperdirect somatosensory activity linking vagal nerve sensory afferents to the brain [9]. The exceptionally rapid neural transmission observed in this response is likely attributable to A-alpha fiber stimulation, whose brain activity may be masked by the stimulus artifact [26]. Furthermore, EVEREST can be recorded ipsilaterally and contralaterally to the stimulation site while maintaining polarity alignment [3-5], in accordance with the gold standard principles for brain somatosensory evoked potential recordings [7,14]. Although this study did not focus on muscle modulation effects on EVEREST, our pilot findings suggest that EVEREST is susceptible to rapid plastic changes following muscle contractions, like those observed in brain somatosensory evoked potentials induced by limb stimulation [27]. This cranial somatosensory neuromodulation likely occurs because muscle contractions reduce neural coherence and disrupt connectivity involved in the cortical processing of somatosensory stimuli within sensorimotor cortices [27-29].

Limitations of the study

First, the sample size was relatively small, which may limit the generalizability of the findings. However, the statistical analyses were robust given this sample size, providing confidence in the accuracy and reliability of the data. Second, while the study focused on specific craniofacial muscles, the findings provide valuable initial evidence that volume conduction may play a role in neural responses. However, future research should investigate additional craniofacial muscles to determine the extent of this effect more comprehensively. Third, the study successfully captured meaningful electromyogram (EMG) activity, supporting the reliability of the recorded neural signals. However, incorporating time-locked EMG activity could enhance temporal precision, allowing for a more detailed examination of the onset and progression of muscle activation. Fourth, although the study examined one muscle at a time, the statistically strong results suggest that including multiple muscles in future research could yield even more robust findings. Fifth, male participants were studied to control for potential variability introduced by hormonal fluctuations, as female sex hormones have been shown to modulate neural responses.

## Conclusions

The fVSEP is an electromyographic response induced both at rest and during subtle, mild, and strong craniofacial muscle contractions. The so-called fVSEP is not, by any means, the electrophysiological representation of tragus sensory afferent pathway activation terminating in the somatosensory homunculus. Instead, it reflects muscle fiber oscillations and motoneuronal recruitment occurring both at rest and during craniofacial muscle activation. In contrast, the EVEREST signals we recorded in the nanosecond range may represent a novel biomarker for monitoring parasympathetic function regulated by auricular neuromodulation in health and disease.
